# Methylenetetrahydrofolate reductase C677T polymorphism in patients with lung cancer in a Korean population

**DOI:** 10.1186/1471-2350-12-28

**Published:** 2011-02-22

**Authors:** Lian-Hua Cui, Min-Ho Shin, Hee Nam Kim, Hye-Rim Song, Jin-Mei Piao, Sun-Seog Kweon, Jin-Su Choi, Woo-Jun Yun, Young-Chul Kim, In-Jae Oh, Kyu-Sik Kim

**Affiliations:** 1 Department of Public Health, Qingdao University Medical College, Qingdao, China; 2 Department of Preventive Medicine, Chonnam National University Medical School, Gwangju, South Korea; 3 Genome Research Center for Hematopoietic Diseases, Chonnam National University Hwasun Hospital, Hwasun, Jeollanam-do, South Korea; 4 Jeonnam Regional Cancer Center, Chonnam National University Hwasun Hospital, Hwasun, Jeollanam-do, South Korea; 5 Lung and Esophageal Cancer Clinic, Chonnam National University Medical School, Hwasun Hospital, Hwasun, Jeollanam-do, South Korea; 6 Yanbian University Medical College, 121 Juzi Street, Yanji, Jilin Province, China

## Abstract

**Background:**

This study was designed to investigate an association between methylenetetrahydrofolate reductase (MTHFR) C677T polymorphism and the risk of lung cancer in a Korean population.

**Methods:**

We conducted a large-scale, case-control study involving 3938 patients with newly diagnosed lung cancer and 1700 healthy controls. Genotyping was performed with peripheral blood DNA for MTHFR C677T polymorphisms. Statistical significance was estimated by logistic regression analysis.

**Results:**

The MTHFR C677T frequencies of CC, CT, and TT genotypes were 34.5%, 48.5%, and 17% among lung cancer patients, and 31.8%, 50.7%, and 17.5% in the controls, respectively. The MTHFR 677CT and TT genotype showed a weak protection against lung cancer compared with the homozygous CC genotype, although the results did not reach statistical significance. The age- and gender-adjusted odds ratio (OR) of overall lung cancer was 0.90 (95% confidence interval (CI), 0.77-1.04) for MTHFR 677 CT and 0.88 (95% CI, 0.71-1.07) for MTHFR 677TT. However, after stratification analysis by histological type, the MTHFR 677CT genotype showed a significantly decreased risk for squamous cell carcinoma (age- and gender-adjusted OR, 0.78; 95% CI, 0.64-0.96). The combination of 677 TT homozygous with 677 CT heterozygous also appeared to have a protection effect on the risk of squamous cell carcinoma. We observed no significant interaction between the MTHFR C677T polymorphism and age and gender or smoking habit.

**Conclusions:**

This is the first reported study focusing on the association between MTHFR C677T polymorphisms and the risk of lung cancer in a Korean population. The T allele was found to provide a weak protective association with lung squamous cell carcinoma.

## Background

Lung cancer is the leading cause of cancer-related death worldwide. The incidence and mortality of lung cancer have been significantly and constantly increasing over the past two decades in Korea [1-3]. According to the Korean National Cancer Registry, the age-standardized incidence rate for lung cancer of Korean population was 47.5/100,000 for men and 13.3/100,000 for women in 2007 [2], it has become the second most common malignant tumor following gastric cancer. The reason for this increase in lung cancer has not been clearly explained. Although it is well known that cigarette smoking is the major cause of lung cancer, only 10-20% of lifetime smokers are known to develop lung cancer. Additionally, lung cancer is a multicellular and multistage process involving a number of genetic changes in oncogenes, suggesting that genetic factors may play an important role in its development [4-6].

Methylenetetrahydrofolate reductase (MTHFR) is an important enzyme in folate metabolism. A common mutation of the MTHFR gene is the C to T transition at nucleotide 677, which converts alanine to valine, results in a thermo-labile enzyme with decreased activity [7]. The heterozygote and homozygous variant of C677T were shown to have 65 and 30% of the enzyme activity, respectively [8]. The low enzymatic activity of the MTHFR C677T genotypic variant is associated with DNA hypomethylation, which may induce genomic instability or the derepression of proto-oncogenes.

To date, several studies have shown that the MTHFR C677T polymorphism are associated with either increased [9-12] or decreased [13-15] risk of lung cancer, whereas others observed no association between the MTHFR C677T genotype and genetic susceptibility to lung cancer [16-18]. Small sample size, various ethnic groups, diet, environment, and methodologies may be responsible for the discrepancy. Therefore, a larger single study is required to evaluate MTHFR C677T polymorphisms and the lung cancer risk in a specific population. Additionally, to our knowledge, no previous report has examined the effect of MTHFR C677T polymorphisms on the risk of lung cancer in a Korean population. In the present study, we performed a large population based case-control study involving 3938 lung cancer patients and 1700 healthy controls to evaluate whether MTHFR C677T polymorphism was associated with lung cancer risk in a Korean population. Additionally, we investigated whether MTHFR C677T plays an interactive role in the lung cancer risk in relation to histological subtypes and smoking status.

## Methods

### Subjects

The study population consisted of 3938 patients with newly diagnosed lung cancer and 1700 population-based controls. All enrolled patients were pathologically confirmed at Chonnam National University Hwasun Hospital between January 2000 and August 2010. Cases with secondary or recurrent tumors were excluded.

The control group (*n *= 1700) consisted of participants in the Thyroid Disease Prevalence Study [19], conducted from July 2004 to January 2006 in the Yeonggwang and Muan Counties of Jeollanam-do Province and in Namwon City of Jeollabuk-do, Korea. A total of 4018 subjects were randomly selected by 5-year age strata and sex. Of the total number, 3486 were eligible subjects. Of those eligible, 1699 (48.8% of the eligible subjects; 820 men and 879 women), underwent clinical examinations. At the time of their peripheral blood collections, all control subjects provided their informed consent to participate in this study.

This study was approved by the Institutional Review Board of the Chonnam National University Hwasun Hospital in Hwasun, South Korea. At the time of their peripheral blood collections, all case and control subjects provided their informed consent to participate in this study.

### Genotyping

Genomic DNA was extracted from peripheral blood using a QIAamp DNA Blood Mini Kit (Qiagen, Valencia, CA, USA) according to the manufacturer's protocol. Genotyping was performed by polymerase chain reaction-restriction fragment length polymorphism (PCR-RFLP) or real-time PCR. The genotyping protocol for PCR-RFLP was adapted from Frosst et al. [8]. After HinfI (Takara, Tokyo, Japan) restriction enzyme digestion, samples were run on a 10% polyacrylamide gel (19:1) using Microtitre Array Diagonal Gel Electrophoresis (MADGE; MadgeBio, Grantham and Southampton, UK). Genotyping by real-time PCR was performed by allelic discrimination using dual-labeled probes containing locked nucleic acids (LNA) in a real-time PCR assay. PCR primers and LNA probes were designed and synthesized by Integrated DNA Technologies (IDT, Coralville, IA, USA). Primers producing a 104-bp amplicon were as follows: forward, 5'-CTTTGAGGCTGACCTGAAGC-3' and reverse, 5'-TCACAAAGCGGAAGAA TGTG-3'. Dual-labeled LNA hybridization probes were 5'-FAM-ATG GcT ccc-BHQ1-3' for the C allele and 5'-cy5-cgA CTc cCg C-BHQ2-3' for the T allele (LNA bases are denoted in upper case, and single nucleotide polymorphisms are underlined). Real-time PCR was performed using a Rotor-Gene 3000 multiplex system (Corbett Research, Sydney, Australia) in a 10-μL reaction volume containing 200 nM PCR primer, 10-10 nM each probe, 0.5 U f-taq polymerase (Solgent, Daejeon, Korea), and 40 ng of genomic DNA. In 24 subjects, the results of PCR-RFLP were compared with those from real-time PCR, and the resulting concordance rate was 100%.

### Statistical analyses

The statistical significance of differences between the patient and control groups was estimated by logistic regression analysis. Adjusted odds ratios (OR) were calculated with a logistic regression model that controlled for gender and age and are given with 95% confidence intervals (CI). Subjects with the wild-type genotypes (MTHFR 677CC) were considered to be at baseline risk. The expected frequency of control genotypes was checked by the Hardy-Weinberg equilibrium test. The heterogeneity was tested by multivariate logistic regression model. Subjects for whom there were missing data for smoking or histological type were excluded in interaction and subgroup analyses related to these variables. All analyses were performed using the Statistical Package for the Social Sciences software (ver. 13.0; SPSS, Chicago, IL, USA).

## Results

The characteristics of the study population are presented in Table 1. In total, 3938 cases and 1700 controls were included in these analyses. The 3938 lung cancer cases consisted of 1523 adenocarcinomas, 1519 squamous cell carcinomas, 574 small cell carcinomas, and 322 other types, including 75 large cell cancers and 247 mixed types. The mean age of patients with lung cancer was significantly higher than the control group. A statistically significant gender difference was also found between patients with lung cancer and healthy controls; the control group had more females. The proportion of smokers in lung cancer cases was higher than in the controls.

**Table 1:** General characteristics of subjects

Characteristics	Cases n (%)	Controls n (%)
**No**.	3938	1700
Age(mean ± SD)*	64.8 ± 9.6	52.2 ± 14.3
≤65 years	1803 (45.8)	1308 (76.9)
> 65 years	2135 (54.2)	392 (23.1)
Gender*		
Male	3128 (79.4)	821 (48.3)
Female	810 (20.6)	879 (51.7)
Smoking status*		
Never	900 (22.8)	1000 (58.8)
Ever	2951 (74.9)	655 (38.5)
unknown	87 (2.2)	45 (2.6)
**Histological type**		
ADC	1523 (38.7)	
SQC	1519 (38.6)	
SCLC	574 (14.6)	
Others	322 (8.2)	

SD, standard deviation; ADC, adenocarcinoma; SQC, squamous cell carcinoma;

SCLC, small cell lung cancer; others, large cell carcinoma and mixed types; *, *p *< 0.01.

Table 2 shows the genotype distributions for MTHFR C677T and their ORs and 95% CIs in lung cancer. The distribution of the MTHFR C677T gene polymorphisms in the controls was calculated by the Hardy-Weinberg equilibrium. The MTHFR C677T frequencies of CC, CT, and TT genotypes were 34.5%, 48.5%, and 17.0% in lung cancer, and 31.8%, 50.7%, and 17.5% in the controls, respectively. The frequencies of combination for 677 CT heterozygous and 677 TT homozygous were observed 65.4% in lung cancer and 68.2% in the controls. Compared with the MTHFR 677 CC genotype, the TT and CT genotypes showed a protective effect for the risk of lung cancer when adjustments were made for age and gender, overall TT versus CC (OR = 0.88; 95% CI = 0.71-1.07) and overall CT versus CC (OR = 0.90; 95% CI = 0.77-1.04); however, the results did not reach statistical significance.

**Table 2:** Distribution of MTHFR C677T and their association with lung cancer risk

MTHFR C677T	Lung cancer n (%)	Control n (%)	OR^a^	95% CI
**CC**	1361 (34.5)	540 (31.8)	1	
**CT**	1909 (48.5)	862 (50.7)	0.90	0.77-1.04
**TT**	668 (17.0)	298 (17.5)	0.88	0.71-1.07
**CT+TT**	2577 (65.4)	1160 (68.2)	0.89	0.78-1.03

^a^Adjusted for age, gender; OR, odds ratio; CI, confidence interval.

Table 3 shows subgroup analysis by gender, age and histological type for the MTHFR C677T polymorphisms. When the MTHFR 677CC genotype was used as the reference group, the MTHFR 677 CT genotype were associated with a significantly reduced risk in squamous cell carcinoma (OR = 0.78; 95% CI = 0.64- 0.96), the combined variant genotypes (677 CT + TT) also showed a protect effect on the risk of squamous cell carcinoma (OR = 0.79; 95% CI = 0.65- 0.95), while there was no significant association in other histological types of lung cancer. There were no heterogeneities among subgroups of gender (male, female), age (age ≤65, age > 65), smoking (never smoker, ever smoker), histological type (adenocarcinoma, squamous cell carcinoma, small cell carcinoma, other types). Nor did we find evidence for an interaction between the MTHFR C677T polymorphisms and age and gender or smoking habit.

**Table 3:** Subgroup analysis for the MTHFR C677T polymorphisms

	CT vs. CC		TT vs. CC		*P*^b^	CT+TT vs. CC		*P*^b^
					
	OR^a^	95%CI	OR^a^	95%CI		OR^a^	95%CI	
**Gender**					0.90			0.96
Male	0.89	0.74-1.08	0.90	0.63-1.16		0.89	0.74-1.07	
Female	0.91	0.72-1.16	0.85	0.63-1.16		0.90	0.72-1.12	
**Age**					0.39			0.84
**≤ **65	0.96	0.80-1.16	0.87	0.68-1.12		0.94	0.79-1.12	
> 65	0.80	0.62-1.04	0.90	0.64-1.25		0.83	0.65-1.05	
**Smoking status**					0.55			0.77
Never	0.90	0.72-1.12	0.80	0.60-1.07		0.87	0.71-1.08	
Ever	0.89	0.73-1.10	0.97	0.73-1.28		0.91	0.75-1.11	
**Histological type**					0.64			0.99
ADC	0.89	0.75-1.05	0.93	0.74-1.16		0.90	0.76-1.06	
SQC	0.78	0.64-0.96	0.80	0.61-1.04		0.79	0.65-0.95	
SCLC	0.96	0.75-1.22	0.83	0.59-1.15		0.92	0.73-1.17	
Others	0.93	0.70-1.26	0.77	0.51-1.15		0.89	0.67-1.18	

SCLC, small cell lung cancer; SQC, squamous cell carcinoma; ADC, adenocarcinoma; others, large cell carcinoma and mixed types.

OR^a^: odds ratio adjusted for age and gender, CI, confidence interval;

*P*^b^: *p *values for heterogeneity.

## Discussion

The current study represents the largest sample (3938 lung cancer patients and 1700 controls) of a single population reported to evaluate a possible association between MTHFR C677T gene polymorphism and susceptibility to lung cancer. To our knowledge, this is also the first report to examine the association between MTHFR C677T polymorphisms and susceptibility to lung cancer in a Korean population. We found that the MTHFR 677 CT and TT showed weak protection for overall lung cancer, although the results were not statistically significant. However, by histological subtype, we found significant protection of the MTHFR CT genotype for squamous cell carcinoma risk.

The combination of 677 TT homozygous with 677 CT heterozygous also appeared to have a protection effect on the risk of squamous cell carcinoma. We observed no significant interactions between the MTHFR C677T polymorphism and smoking, gender, or age.

Results of several studies examining the role of the MTHFR C677T polymorphism in lung cancer susceptibility have been inconsistent. Liu et al. [14] and Jeng et al. [13] in Taiwan and Suzuki et al. [15] in Japan showed that the MTHFR 677 TT genotype was associated with a decreased risk of lung cancer. However, Siemianowicz et al. [11] in Poland, Hung et al. [9] in Central Europe, and Shen et al. [10] in China showed that individuals with MTHFR TT genotype had an increased risk of lung cancer versus those with the wild-type homozygous variant, while a recent meta-analysis by Mao et al. [20] based on eight case-control study suggested no evidence for a major role of the MTHFR C677T polymorphisms in carcinogenesis of lung cancer. Small sample size, various ethnic groups, diet, environment, and methodologies might be responsible for the discrepancy.

The pathogenesis of adenocarcinoma is considered to be somewhat different from that of squamous cell carcinomas, and whether the effect of MTHFR C677T polymorphism differs by lung cancer histology remains unclear. We performed a stratification analysis by histological type, which is lacking in most previous studies, and found that the MTHFR 677 CT genotype was associated with a significantly decreased risk for lung squamous cell carcinoma (OR = 0.78, 95%CI = 0.64-0.96), supporting the potential effect of MTHFR C677T polymorphism on lung squamous cell carcinoma. In other histological type of lung cancer, such as adenocarcinoma and small cell lung cancer, we found no association between C677T MTHFR genotype and lung cancer risk. A similar result was seen in a Japanese study in which no effect of MTHFR C677T polymorphism on the risk of overall lung cancer was evident, but on histology based analysis, the MTHFR 677T allele was associated with a reduced risk of squamous/small cell carcinoma [15]. While Siemianowicz et al. [11] reported that the 677TT genotype was associated with a significantly higher risk of non-small cell lung cancer.

The role of MTHFR polymorphisms in modulating cancer risk is associated with folate status. Under adequate folate conditions, the protective effect of the 677TT genotype turns to a situation of elevated risk of lung cancer among MTHFR 677TT genotype with low folate intakes. A recent meta-analysis by Boccia et al. [21], which included stratified analysis according to dietary folate intake, showed an increased risk for individuals with low folate intake (OR = 1.28, 95% CI = 0.97-1.68 for lung) versus high folate intake (OR = 0.94, 95% CI = 0.79-1.12 for lung). Plasma folate level might be relatively high among Korean adults; the median plasma folate was 22.7 nmol/L in our population based controls, which is higher than that in Chinese population [22], and also higher than that in populations from15 European countries (folate status ranged from 6.3 to 20.1 nmol/L) [23]. In addition, folate intake seems also fairly high among Korean population; the average of folate intake in Korea is about 347 μg/day [24]. This level is higher than the average intake in most European countries, except for United Kingdom [23]. This might provide a partial explanation why the MTHFR 677 mutations were found to protect against lung cancer, especially in lung squamous cell carcinoma in our study. Moreover, our recent study found a protective effect of MTHFR 677 T allele on the risk of gastric and colorectal cancer in a Korean population [25].

In our study, there was no significant gender difference in the effect of MTHFR C677T polymorphism on lung cancer risk. Our results seem to differ from those of the Shi et al. [26] study in Houston, TX, USA, which reported that the MTHFR 677TT genotype in women was associated with a decreased lung cancer risk compared with carriers of the MTHFR 677CC genotype.

Cigarette smoking is a known risk factor for lung cancer, and smokers may tend to have lower levels of serum and produce a localized deficiency of folic acid. We further examined the effects of MTHFR C677T in subgroups according to smoking status and found no interaction between the MTHFR C677T polymorphism and smoking. Our results seem somewhat similar to the results of Vineis, et al. [18], which showed that the MTHFR C677T polymorphism had no any association in both smokers and nonsmokers. However, a beneficial effect of the MTHFR TT genotype on the risk of lung cancer was observed in those with heavy smokers; Suzuki et al. [15] in Japan found that MTHFR 677T alleles were associated with reduced risk of squamous/small cell carcinomas, especially among heavy smokers with the MTHFR 677T allele. Liu et al. [14] in Taiwan observed that smokers carrying the MTHFR 677 T allele showed a significantly decreased risk of lung cancer.

It is well known that familial aggregation of lung cancer could increase the risk of lung cancer, and a high consumption of vegetables and fruits is associated with a reduced risk of lung cancer. However, we have no information on the accuracy of reported family history of cancer, dietary folate intake or detailed data on the environmental tobacco exposure risk factors for lung cancer. Thus, we cannot evaluate the relationship between gene-environment interactions. Another limitation of the present study is that the case group was composed of lung cancer patients who were enrolled from hospital, which could not be representative the general population.

## Conclusions

Our present large case-control study in Korea found a protective effect of the MTHFR C677T variant genotype for lung squamous cell carcinoma and suggested that the effects of MTHFR C677T polymorphism may be involved in the development of lung cancer for Korean population.

## Competing interests

The authors declare that they have no competing interests.

## Authors' contributions

MHS planned the analysis. CLH performed in the study design and drafted the manuscript. HNK and HRS participated in the experiments. JMP performed data analysis. YCK, IJO and KSK provided clinical material. SSK, JSC, and WJY participated in its design and coordination. All authors read and approved the final manuscript.

## Pre-publication history

The pre-publication history for this paper can be accessed here:

http://www.biomedcentral.com/1471-2350/12/28/prepub
